# The Ecological, Biological, and Social Determinants of Dengue Epidemiology in Latin America and the Caribbean: A Scoping Review of the Literature

**DOI:** 10.1007/s10393-025-01706-0

**Published:** 2025-03-27

**Authors:** Aisha Barkhad, Natacha Lecours, Maya Stevens-Uninsky, Lawrence Mbuagbaw

**Affiliations:** 1 Department of Global Health, McMaster University, 1280 Main St W, Hamilton, ON L8S 4L8 Canada; 2 Global Health Division, International Development Research Centre (IDRC), Ottawa, Canada; 3 Department of Health Research Methods Evidence and Impact, McMaster University, Hamilton, Canada; 4 Department of Anesthesia, McMaster University, Hamilton, ON Canada; 5 Department of Pediatrics, McMaster University, Hamilton, ON Canada; 6 Biostatistics Unit, Father Sean O’Sullivan Research Centre, St Joseph’s Healthcare, Hamilton, ON Canada; 7 Centre for Development of Best Practices in Health (CDBPH), Yaoundé Central Hospital, Yaoundé, Cameroon; 8 Division of Epidemiology and Biostatistics, Department of Global Health, Stellenbosch University, Cape Town, South Africa

**Keywords:** Dengue, Latin America, Caribbean, Epidemiology, Eco-bio-social, Scoping review

## Abstract

Dengue has re-emerged in Latin America and the Caribbean (LAC) over the last five decades. The factors influencing dengue transmission by the *Aedes aegypti* mosquito vector within the region can be classified as ecological, biological, and social determinants. In this review, we summarized the published literature on the evidence for the determinants of dengue vector dynamics, transmission, and epidemiological outcomes in LAC. We searched PubMed, SCOPUS, and LILACS databases in September 2022 to collect published works irrespective of study design published in either English, French, Portuguese, or Spanish. Full-text articles were obtained for the studies that passed the title and abstract screening process. During full-text screening, articles were assessed to determine if they met the eligibility criteria. Data were extracted using NVivo™ 12. Data were organized as NVivo codes. Themes were compiled and communicated narratively. We included 90 peer-reviewed research articles from LAC between 2007 and 2022. The included studies were from 15 different countries, dependencies, and territories in the region. Several dengue-related indicators and outcomes were classified as ecological, biological, or social. Eight main factors were found, including: micro- and macro-climatic factors; entomological and pathogenic factors; and global-, community-, household-, and individual- level social factors. We identified several existing knowledge gaps in the literature. Making salient these gaps may serve as a starting point for future vector-borne infectious disease research to equip policymakers and public health practitioners to develop effective strategies to mitigate the impact of dengue and protect vulnerable populations in LAC.

**Supplementary Information:**

The online version contains supplementary material available at 10.1007/s10393-025-01706-0.

## Introduction

Dengue is the most common and most widespread human arboviral disease in the world (Bhatt et al., 2013). Transmissible by the *Aedes aegypti* and the *Aedes albopictus* mosquito vectors, the dengue virus (DENV), in all its four serotypes, represents a major public health threat in many parts of Mesoamerica and South America (Gómez-Dantés & Willoquet, 2009). Dengue has re-emerged in Latin America and the Caribbean (LAC) over the last 50 years due to the re-infestation of the *A. aegypti* mosquito in the region (Zambrano & San Martin, 2014). Recently, documented dengue cases in LAC have increased as epidemics have become widespread (Tapia-Conyer et al., 2012).

The frequency of dengue epidemics is influenced by seasonality and weather patterns (Morin et al., 2013), especially anomalies of temperature, rainfall, and relative humidity. Seasonality is shaped by the Earth’s inter-annual climate cycle on a global scale, and patterns of seasonal dengue epidemics are correlated with the El Niño Southern Oscillations (ENSO; Gagnon et al., 2001). ENSO is a natural cycle, which is a coupled atmospheric-oceanic system that produces short-term climate and sea surface temperature changes over the Pacific region and has implications for dengue outbreaks (McGregor & Ebi, 2018).

The intrinsic entomological and pathogenic characteristics of the dengue vector and virus also have a regulatory role in DENV outbreaks (Jones et al., 2008). Importantly, vectorial capacity and competence of *A. aegypti* represents the vector’s propensity and ability to acquire, maintain, and transmit DENV and has consequences for dengue transmission (Liu-Helmersson et al., 2014). Vectorial capacity and competence are regulated by genetic variabilities phenotypically expressed by not only the vector, but also the virus, and can vary depending on infection by distinct DENV serotypes (Severson & Behura, 2016).

Urbanization is a social driver of DENV transmission in LAC and is contributing to increased human population densities in already crowded cities, providing opportunities for DENV transmission within human settlements (Gubler, 2011). Disorderly urban planning also promotes the growth of ‘poverty pockets’ and may increase the risk of DENV among populations (Gómez-Dantés & Willoquet, 2009; Tapia-Conyer et al., 2012). Poor intra- and peri-domiciliary housing conditions including inadequate structures (e.g., roofs, walls, and flooring), have been associated with DENV transmission (Coreil et al., 2013). Additionally, human water-storing behaviors, which facilitate superlative breeding sites for mosquito vectors, are an important social determinant of DENV outbreak potential and are associated with access to reliable water sources in communities (Gibson et al., 2014).

Since viable and universal vaccination campaigns have not yet been launched within the LAC region, efforts to contain DENV epidemics typically rely on community-based vector control and prevention strategies. Understanding the factors affecting the patterns of DENV epidemics in LAC is a challenge and may allow for targeted prevention campaigns and vaccine introduction. This challenge underscores the need for identifying the distinct factors impacting dengue in the LAC region to provide the groundwork for prospective research and policy for DENV prevention and control. Therefore, the aim of this scoping review is to summarize the published literature on the ecological, biological, and social determinants of DENV vector dynamics, transmission, and epidemiological outcomes in LAC.

## Methodology

### Registration

This review was registered in the Open Science Framework (OSF) Registries (DOI: https://doi.org/10.17605/OSF.IO/9Z268).

### Eligibility Criteria

Research articles published between 2007 and 2022, inclusively, were sought to capture the interval following the release of the Intergovernmental Panel on Climate Change (IPCC) 4th Assessment Report (IPCC, 2007). We included qualitative, quantitative, and mixed methods studies; short communications of findings; and discussion papers set in LAC, in either English, French, Portuguese, or Spanish. We excluded books and book chapters, systematic reviews, meta-analyses, pre-print articles, and Gray literature (Table S1).

### Data Sources and Search Strategy

We searched PubMed, SCOPUS, and Latin American & Caribbean Health Sciences Literature (LILACS) in September 2022. The literature search strategies were developed using medical subject headings (MeSH) and relevant keywords adapted to each of the databases. See the Supplemental Material for search terms used. Additional articles were compiled from bibliographic references of included articles (Table S2, S3, S4).

### Data Management and Selection

A two-stage screening procedure was implemented to select studies. Searches were conducted in the databases, and retrieved records were managed in Zotero software 5.0.67. Published works were transferred to Covidence (Veritas Health Innovation, Melbourne, Australia). Full-text articles were obtained for the studies that passed the title and abstract screening process. The full-text articles were assessed to determine whether they met the eligibility criteria. Articles that met the criteria were transferred to NVivo™ 12 for data analysis (QSR International, Cambridge, USA). Figure S1 shows the PRISMA flowchart describing the search strategy and screening procedure.

### Data Collection and Analysis

Data from full-text articles were coded in NVivo™ 12. Codes were highlighted to sort ecological, biological, and social factors and themes. A Codebook was developed and rules for coding were established. A data extraction form in Microsoft Excel 2019 (Version 16) was used to obtain information on the characteristics of the articles (i.e., country, year of publication, journal, etc.) and the context of the article (i.e., study methods, indicators, outcomes, etc.; Table S5).

## Results

### Characteristics of Included Studies

**Figure 1:**
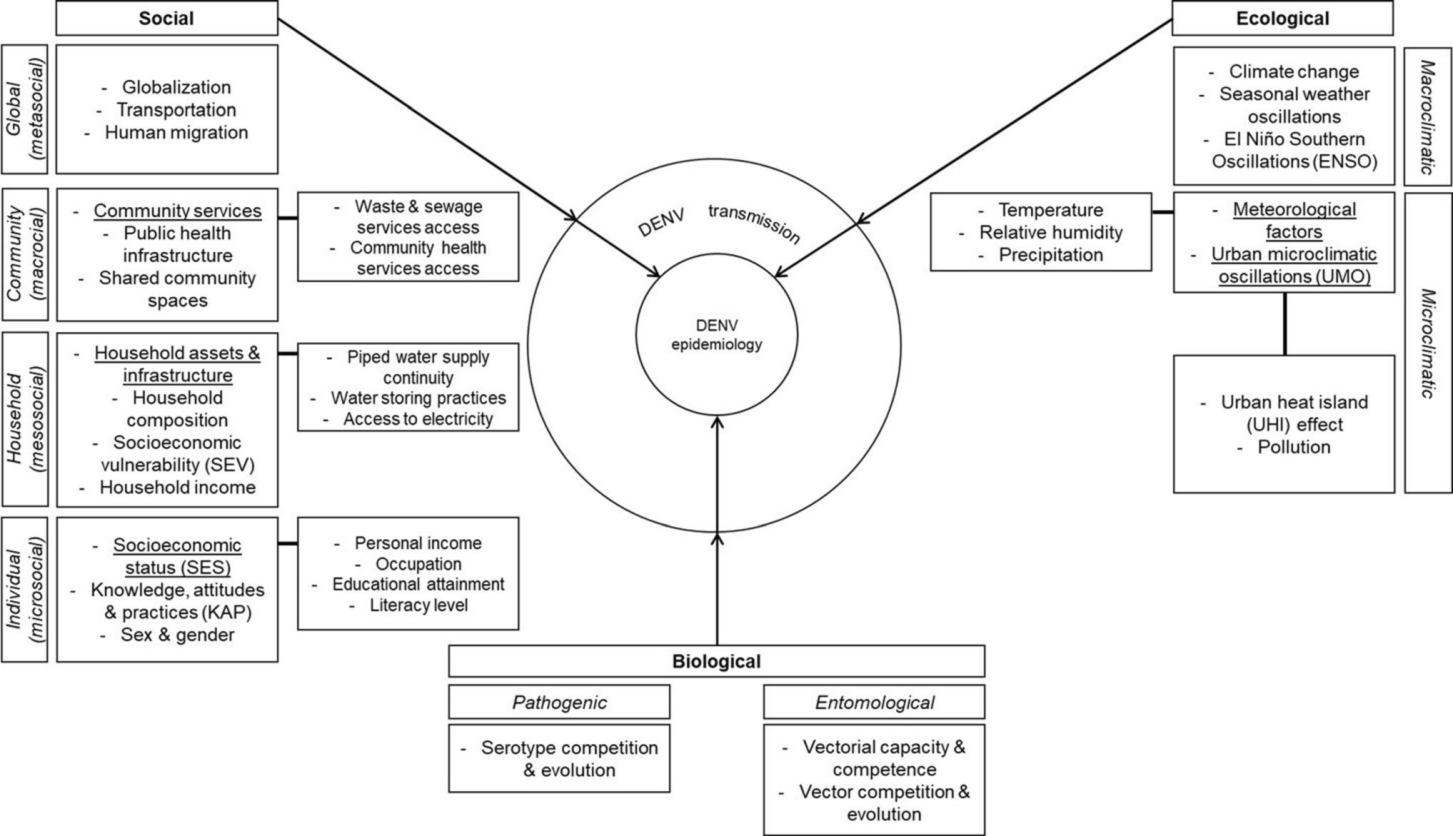
Ecological, biological, and social factors determining dengue virus transmission and epidemiological outcomes in LAC, based on a scoping review of the literature.

We included 90 articles in this review from 15 different countries, dependencies, and/or territories from LAC (Table 1). Of these, nearly three quarters were from South America (n = 66), whereas nearly one quarter of the studies were from Central America and the Caribbean Islands (n = 13 and n = 6, respectively). Other studies were conducted in multiple countries (n = 2) or were classified as laboratory-based studies (n = 3). Most studies were from Brazil (n = 36) and Colombia (n = 12). Fewest studies were published in 2007 (n = 1). Most studies were published in 2021 (n = 11). The included research papers were classified as: ecological, biological, or social studies (Table 2). Most studies were classified as social studies (n = 39), followed by biological studies (n = 28), and ecological studies (n = 23). Eight main factors influencing DENV vector dynamics, transmission, and epidemiological outcomes in LAC were identified and are illustrated in Fig. 1.

**Table 1:** List of studies included in a scoping review of the literature on the ecological, biological, and social determinants of dengue vector dynamics, transmission, and epidemiology in Latin America and the Caribbean.

Author(s), Year of publication	Study setting	Study design	Indicator(s)*	Outcome(s)	Factor
Alto et al. (2008a)	N/A	Experimental study	Vector competition	DENV infection, vector competence	Biological
Alto et al. (2008b)	N/A	Experimental study	Vector physiology	DENV infection	Biological
Aponte et al (2013)	Mexico	Experimental study	Exposure to insecticide	Vector insecticide resistance status	Biological
Arboleda et al (2012)	Colombia	Ecological niche modeling	Vector presence	DENV incidence	Biological
Barbosa et al (2014)	Brazil	Spatial case–control study	Vector presence	DENV risk	Biological
Bellinato et al (2016)	Brazil	Experimental study	Exposure to insecticide	Vector insecticide resistance status	Biological
Bennett et al (2021)	Panama	Experimental study, genetic analyses	Temperature, vegetation, rainfall humidity	Vector adaptability	Biological
Burke et al (2010)	Puerto Rico	Observational study	Vector larval presence	Vector abundance	Biological
Carreño et al (2019)	Colombia	Experimental study, genetic analyses	DENV serotype changes	DENV cases	Biological
Carrington et al (2013)	N/A	Experimental study	Temperature	Vector lifecycle	Biological
Cromwell et al (2017)	Peru	Entomological, longitudinal epidemiological study	Vector density	DENV seroconversion	Biological
Dusfour et al (2011)	French Guiana	Experimental study	Exposure to insecticide	Vector insecticide resistance status	Biological
Flores et al (2013)	Mexico	Experimental study	Exposure to insecticide	Vector insecticide resistance status	Biological
Francis et al (2020)	Jamaica	Experimental study	Exposure to insecticide	Vector insecticide resistance status	Biological
Gonçalves et al (2014)	Brazil	Experimental study	Experimental DENV infection	Vector competence	Biological
Gustave et al (2012)	Guadeloupe	Observational study	Vector breeding site presence	Vector abundance	Biological
Honório et al (2009)	Brazil	Spatial modeling study	Vector density	DENV seroprevalence	Biological
Lima et al (2011)	Brazil	Experimental study	Exposure to insecticide	Vector insecticide resistance status	Biological
Lima Júnior & Scarpassa (2009)	Brazil	Experimental study, phylogenetic analysis	Distinct vector populations	Vector genetic variability	Biological
Lourenço-de-Oliveira et al (2013)	Multi-country	Experimental study	Experimental DENV infection	Vector competence	Biological
Marcombe et al (2009)	Martinique Islands	Experimental study	Exposure to insecticide	Vector insecticide resistance status	Biological
OhAinle (2011)	Nicaragua	Experimental study, phylogenetic analysis	DENV genetics	DENV cases	Biological
Rahman et al (2021)	Brazil	Experimental study, genetic analyses	Exposure to insecticide	Vector insecticide resistance status	Biological
Sá et al (2019)	Brazil	Experimental study	Exposure to insecticide	Vector insecticide resistance status	Biological
Santiago et al (2012)	Puerto Rico	Experimental study, phylogeographic analysis, evolutionary analysis	DENV genetics	DENV evolution	Biological
Solis-Santoyo et al (2021)	Mexico	Experimental study, spatial study	Exposure to insecticide	Vector insecticide resistance status	Biological
Vazeille et al (2016)	French Guiana	Experimental study	Vector competence	DENV serotype competition	Biological
Whiteman et al (2019)	Panama	Spatial temporal modeling study	Vector infestation	DENV prevalence	Biological
Amarakoon et al (2008)	Multi-country	Epidemiological study	Temperature, precipitation	DENV incidence	Ecological
Araujo et al (2015)	Brazil	Ecological study, epidemiological study	Temperature, vegetation, population density, SES, housing conditions	DENV incidence	Ecological
Campbell et al (2015)	Peru	Epidemiological study	Temperature, humidity	DENV incidence, DENV transmission potential	Ecological
Carneiro et al (2017)	Brazil	Epidemiological study, cross-sectional observational study, ecological planning model, temporal trend analysis	Temperature, pollution	DENV incidence	Ecological
Chowell et al (2011)	Peru	Epidemiological study, temporal trend analysis	Temperature, precipitation	DENV incidence	Ecological
Dostal et al (2022)	Peru	Ecological study, Time series study	Temperature, precipitation, ENSO events	DENV cases	Ecological
Duarte et al (2019)	Brazil	Ecological study, epidemiological study	Temperature, precipitation, relative humidity, river level	DHF incidence	Ecological
Fuller et al (2009)	Costa Rica	Climatological modeling study	Sea surface temperatures, vegetation	DHF/DF incidence	Ecological
Gomes et al (2012)	Brazil	Epidemiological study, analytical ecological design	Temperature, precipitation	DENV cases	Ecological
Hurtado-Díaz et al (2007)	Mexico	Ecological study, time series analysis	Temperature, precipitation, ENSO events	DENV incidence	Ecological
Marinho et al (2022)	Brazil	Environmental observational study, temporal analysis	Temperature, precipitation, ENSO events	DENV incidence	Ecological
Morin et al (2022)	Brazil	Ecological study, epidemiological study	Temperature, air pressure, specific humidity, relative humidity	DF incidence	Ecological
Muñoz et al (2021)	Colombia	Ecological study, epidemiological study, temporal analysis study	Temperature, precipitation, wind velocity, relative humidity, ENSO events	DENV incidence	Ecological
Navarro Valencia et al (2021)	Panama	Epidemiological study, time series analysis	Temperature, precipitation, relative humidity	DENV incidence	Ecological
Peña-García et al (2017)	Colombia	Epidemiological study	Temperature	DENV incidence	Ecological
Pereira da Silva et al (2022)	Brazil	Ecological study, epidemiological study	Vegetation indices	DENV cases	Ecological
Quintero-Herrera et al (2015)	Colombia	Epidemiological study	Temperature, precipitation, ENSO events	DENV incidence	Ecological
Silva et al (2015)	Brazil	Epidemiological study, temporal trend analysis	Temperature, precipitation, relative humidity	DENV incidence	Ecological
Silva et al (2016)	Brazil	Ecological study, epidemiological study, temporal analysis	Temperature, precipitation	DENV incidence	Ecological
Troyo et al (2009)	Costa Rica	Ecological study, epidemiological study	Temperature, precipitation, vegetation indices, environmental built area	DENV incidence	Ecological
Vincenti-Gonzalez et al (2018)	Venezuela	Epidemiological study, Time series analyses	ENSO events	DENV incidence	Ecological
Xavier et al (2021)	Brazil	Epidemiological study, time series analysis	Temperature, precipitation	DENV incidence	Ecological
Zambrano et al (2012)	Honduras	Epidemiological study, Multiple linear regression model	Temperature, precipitation, relative humidity, ENSO events	DHF incidence	Ecological
Barrera et al (2021)	Puerto Rico	Entomological study, spatial analysis	Vector productivity per household, location	Vector presence	Social
Bavia et al (2020)	Brazil	Epidemiological study, spatiotemporal study	Temperature, rainfall, income	DENV incidence	Social
Benítez-Díaz et al (2020)	Colombia	Epidemiological study, Nested cross-sectional analytical study, cohort study	Age, gender, education, number of persons in household, household income, KAP	DENV risk perception	Social
Braga et al (2010)	Brazil	Epidemiological study	Age, sex, education, household characteristics, access to water supply, garbage collection	DENV seroprevalence	Social
Campos et al (2021)	Brazil	Ecological study, epidemiological study	Sex, age, precipitation, temperature, humidity, health vulnerability index	DENV incidence rate	Social
Carabalí et al (2017)	Colombia	Epidemiological, community-based study	Sex, age, ethnicity	DENV seroprevalence, DENV seroconversion	Social
Castro-Bonilla et al (2018)	Colombia	Cross-sectional, epidemiological study	KAP, sewer connection, access to water supply, toilet discharge services, garbage collection, education, employment status, age	DENV seropositivity	Social
Charette et al (2020)	Peru	Epidemiological study	Age, gender, district	DENV incidence	Social
Chiaravalloti-Neto et al (2019)	Brazil	Epidemiological, cohort study	Sex, race, occupation, education, household type, household ownership, number of members	DENV seroprevalence	Social
da Conceição Araújo et al (2020)	Brazil	Ecological study, spatiotemporal modeling study	Income, education, access to water supply, household density, sewage connection, literacy, poverty	DENV incidence rate	Social
da Silva-Nunes et al (2008)	Brazil	Epidemiological study	Household structure, access to water supply, wealth, sex, age, land tenure	DENV seroprevalence, DENV seroconversion	Social
do Carmo et al (2020)	Brazil	Ecological study, spatiotemporal modeling study	Population density, education, literacy, income, access to water supply, access to electricity, social vulnerability	DENV incidence rate	Social
Farinelli et al (2018)	Brazil	Ecological, spatiotemporal study	HOH income, HOH sex, household income, people per household, HOH literacy	DF risk	Social
Ferreira et al (2021)	Brazil	Epidemiological, cross-sectional study	Type of house, access to water supply, garbage collection, education, sex, age, race	DENV seroprevalence	Social
Flauzino et al (2009)	Brazil	Epidemiological study	Age, sex, education, access to water supply, garbage collection	DF incidence	Social
Honorato et al (2014)	Brazil	Ecological study, spatial modeling study	Literacy, access to water supply, garbage disposal, income	DENV incidence	Social
Johansen et al (2018)	Brazil	Epidemiological study, geospatial study	Proximity to breeding sites, garbage collection, sewage connection, access to water, household income, race, household ownership	DF incidence	Social
Kalbus et al (2021)	Brazil	Ecological study, epidemiological study	Household income, poverty, sanitation, healthcare access	DF incidence	Social
Kenneson et al (2017)	Ecuador	Epidemiological study	HOH age, HOH sex, HOH education, HOH employment, people per house, beds per house, people per bedroom, patio, household ecological characteristics, air conditioning, sewage connection, water storage	DENV infection	Social
Kikuti et al (2015)	Brazil	Epidemiological, geospatial study	Proximity to healthcare center, poverty, household population density, race, literacy, sewage connection, access to water supply, garbage collection	DENV infection	Social
Lippi et al (2021)	Ecuador	Epidemiological study, entomological study	Housing condition indicators, household demographics and practices	DENV seropositivity/infection	Social
Lippi et al (2018)	Ecuador	Epidemiological study, geospatial study	Household conditions, age, education, employment, sex	DENV severity of outbreaks/burden, DENV hotspots	Social
Maccormack-Gelles et al (2018)	Brazil	Epidemiological study, spatiotemporal study	Access to electricity, access to water supply, sewage connection, garbage collection, household population density, household income	DENV cases	Social
Maljkovic Berry et al (2020)	Ecuador	Experimental study, phylogeographic analysis, evolutionary analysis	Immigration	DENV transmission	Social
Martínez-Vega et al (2015)	Mexico	Epidemiological study, prospective cohort study	Household location, toilet discharge, access to water supply, SES, insecticide use, household characteristics, individual-level characteristics	DENV infection	Social
Nunes et al (2014)	Brazil	Experimental study, phylogeographic analysis, evolutionary analysis, spatiotemporal analysis	Vector infestation index, number of scheduled flights, population density	DENV transmission	Social
Padmanabha et al (2015)	Colombia	Epidemiological study, spatial analysis	Human movement	DENV transmission	Social
Quinteiro et al (2009)	Colombia	Epidemiological study, cross-sectional study	Gender, education, occupation, SES, health insurance, KAP	Vector dynamics	Social
Reiner Jr. et al (2014)	Peru	Epidemiological study, modeling study	Human movement	DENV infection	Social
Shragai et al (2022)	Colombia	Retrospective geospatial analysis	Public transit usage, public transit lines, socioeconomic status, human mobility	DENV risk	Social
Shuaib et al (2010)	Jamaica	Descriptive study, cross-sectional study	Gender, age, education, occupation, marital status	DENV KAP	Social
Stewart Ibarra et al (2013)	Ecuador	Entomological, epidemiological study	Water storage practices, access to water supply, house condition, knowledge, perceptions of dengue	Vector dynamics	Social
Stewart Ibarra et al (2014)	Ecuador	Qualitative study, focus group discussions	Prevention practices, perceptions, HOH education, HOH sex, HOH employment, garbage collection, sewage connection, access to water supply	DENV perceptions	Social
Stoddard et al (2013)	Peru	Epidemiological study, case–control study, spatial analysis	Human movement, DENV attack rate	DENV incidence	Social
Teixeira & Cruz (2011)	Brazil	Epidemiological study, spatial modeling study	Precipitation; Breteau index; social development index, municipal human development index, income	DENV incidence	Social
Vargas et al (2015)	Brazil	Ecological study, epidemiological study	Access to water supply, garbage collection, sex, literacy, household location	DENV incidence, DENV risk	Social
Vásquez-Trujillo et al (2021)	Colombia	Epidemiological study, observational cross-sectional study	Income, property condition, water supply, garbage collection, internet, number of people per house, number of rooms	Vector dynamics	Social
Velasco-Salas et al (2014)	Venezuela	Cross-sectional, epidemiological study	SES, household size, household ownership, presence of water containers, use of insecticides, access to water supply	DENV seroprevalence	Social
Vincenti-Gonzalez et al (2017)	Venezuela	Epidemiological study, community-based cross-sectional study	SES, household crowding, occupation, number of rooms, persons per household, presence of vector breeding sites, age, sex	DENV seroprevalence	Social

^ǂ^DENV, dengue virus; DF, dengue fever; DHF, dengue hemorrhagic fever; HOH, household head; KAP, knowledge, attitudes, and practices; SES, socioeconomic status; SEV, socioeconomic vulnerability; TDS, total dissolved solids.

**Table 2:** Descriptive statistics of the included studies.

	Description	Number of studies (n, %)
Region	South America	66 (73.3%)
	Central America	13 (14.4%)
	Caribbean Islands	6 (6.7%)
	Laboratory-based	3 (3.3%)
	Multi-country	2 (2.2%)
Country	Brazil	36 (40.0%)
	Colombia	12 (13.3%)
	Peru	7 (7.8%)
	Ecuador	6 (6.7%)
	Mexico	5 (5.6%)
	Panama	3 (3.3%)
	Puerto Rico	3 (3.3%)
	Venezuela	3 (3.3%)
	Laboratory-based	3 (3.3%)
	Costa Rica	2 (2.2%)
	French Guiana	2 (2.2%)
	Jamaica	2 (2.2%)
	Multi-country	2 (2.2%)
	Guadeloupe	1 (1.1%)
	Honduras	1(1.1%)
	Martinique Islands	1 (1.1%)
	Nicaragua	1 (1.1%)
Year	2007	1 (1.1%)
	2008	5 (5.6%)
	2009	7 (7.8%)
	2010	3 (3.3%)
	2011	5 (5.6%)
	2012	5 (5.6%)
	2013	6 (6.7%)
	2014	7 (7.8%)
	2015	8 (8.9%)
	2016	3 (3.3%)
	2017	6 (6.7%)
	2018	6 (6.7%)
	2019	5 (5.6%)
	2020	7 (7.8%)
	2021	11 (12.2%)
	2022	5 (5.6%)
Classification	Ecological	23 (25.6%)
	Biological	28 (31.1%)
	Social	39 (43.3%)

### Ecological Factors (23 Studies)

#### Microclimatic Factors

Meteorological Factors.

Some studies reported a strong relationship between minimum, maximum, and mean temperatures and the occurrence of epidemics and transmission potential of DENV, where higher temperatures were associated with increased DENV incidence (Amarakoon et al., 2008; Peña-García et al., 2017). Relative humidity revealed similar effects as temperature on DENV occurrence (Zambrano et al., 2012). Spatiotemporal and climate-based modeling studies have suggested that levels of precipitation can predict the timing of DENV outbreaks (Duarte et al., 2019), where DENV epidemics peaked most frequently with increased abundant rainfall, followed by a time lag representative of the period between vector breeding and life cycle development, and human infection (Troyo et al., 2009; Chowell et al., 2011; Silva et al., 2015; Xavier et al., 2021).

Artificial Environmental Factors.

One study conducted by Araujo et al., (2015) showed that the urban heat island (UHI) effect and the urban environment favor the transmission of DENV in São Paulo, Brazil. Other researches from urban regions of LAC have cited the role of this phenomenon (Vincenti-Gonzalez et al., 2018); however, less is understood about the magnitude of this relationship. Heatwaves in UHI environments increase concentrations of air-borne particulates. One study found that lower particulate matter (PM_10_) values were associated with higher DENV cases in Brazil (Carneiro et al., 2017).

#### Macroclimatic Factors

Climatological and Seasonal Factors.

The dynamics of DENV transmission in endemic and hyperendemic areas was compounded by local seasonal weather oscillations (Morin et al., 2022; Zambrano et al., 2012). El Niño is correlated with negative (i.e., cooler) anomalies of precipitation, soil moisture and river flows, and positive (i.e., warmer) air temperature anomalies, whereas the opposite is true for the La Niña cool phase (Vincenti-Gonzalez et al., 2018). In Costa Rica, La Niña was more likely to favor greater numbers of DENV fever cases, since this region is linked to increased levels of precipitation in La Niña years (Fuller et al., 2009). This finding is similar to studies from Brazil (Marinho et al., 2022) and Colombia (Quintero-Herrera et al., 2015; Muñoz et al., 2021). El Niño years were associated with increased DENV cases in Mexico (Hurtado-Díaz et al., 2007), despite this region being prone to droughts and dry periods during warm phase years. Similar findings were documented in Peru (Dostal et al., 2022).

### Biological Factors (28 Studies)

#### Entomological Factors

Vectorial Capacity and Competence.

Laboratory-based studies have documented genetically based differences in DENV vectorial competence in *A. aegypti* populations within and between geographic areas (Gonçalves et al., 2014). For example, the body size of the female *A. aegypti* mosquito, determined by its genetic make-up, was suspected to be an important contributing factor to vectorial capacity (Alto et al., 2008a). One experimental study that investigated vectorial competence discovered greater risks for the establishment of the disease in novel regions. Lourenço de Oliveira et al*.* (2013) demonstrated that *A. aegypti* populations from Uruguay, which is a country without any local DENV transmission, were found to be competent in transmitting DENV.

Vector Competition and Evolution.

Alto et al. (2008b) showed that high levels of intraspecific or interspecific competition among larvae enhanced the susceptibility of *A. albopictus* to DENV infection and increased the potential for viral transmission. Both DENV adult mosquito vectors can coexist and persist together. However, competitive interaction between *Aedes* species mosquitoes is possible and is dependent on the environmental conditions of the landscape and the level of urbanicity in the region. For instance, one study recorded a displacement of *A. aegypti* for *A. albopictus* under suboptimal, wet, tropical climate conditions, and more vegetated environments in Panama (Bennett et al., 2021).

#### Pathogenic Factors

Serotype Competition and Evolution.

The competitive displacement of DENV serotypes within vectors from distinct geospatial areas has been documented (Carreño et al., 2019). In French Guiana, predominant lineage change events were identified resulting in the competitive displacement of DENV-1 to DENV-4, which may be explained by deleterious genetic mutations of DENV (Vazeille et al., 2016). Additionally, a phylogeographic analysis from Puerto Rico, combined with serotype-specific incidence data from the region, showed that the transmission of a DENV serotype at geographic and temporal scales was correlated with the absence of other serotypes (Santiago et al., 2012). Serotype competition and the dominance of individual serotypes among human populations may be influenced by human immunological profiles due to cross-reactive humoral and cellular immune responses (Reiner Jr. et al., 2014). Transient serotype cross-protection may have consequences for the clustering of phylogenetically related viruses and serotype exclusion, dominance, and overall frequencies, as evidenced in Nicaragua for distinct DENV serotypes and specific DENV-2 genotypes (OhAinle et al., 2011).

### Social Factors (39 Studies)

#### Global-Level Factors

Globalizing forces, including the transportation of humans and vectors, may determine DENV transmission. For example, one study demonstrated the role of aerial transportation of humans and vectors in DENV transmission in Brazil (Nunes et al., 2014). Human migration is another globalizing force, where the movement or forced displacement of individuals may encourage the introduction of DENV to novel locations. For example, the migration of Venezuelan and Colombian refugees to Ecuador in 2011 and 2013 contributed to the introduction of DENV-1 and DENV-2 serotypes to the region, according to a phylogeographic analysis (Maljkovic Berry et al., 2020).

#### Community-Level Factors

Community-level determinants of DENV infection from the literature included inadequate sewage connection, weekly domestic solid waste collection, and garbage disposal (Honorato et al., 2014; Castro-Bonilla et al., 2018; Lippi et al., 2021), which create favorable conditions for peridomicile and intradomicile *A. aegypti* infestation and greater possibilities for DENV infection among community members. Evidence also suggested that the proximity of communities to important municipal infrastructural areas and shared services was associated with DENV outcomes. For instance, in Itaboraí, Rio de Janeiro, Brazil, regions located along major municipal highways with increased vehicular traffic were at higher risk of DENV infections (Vargas et al., 2015). Similarly, municipalities located near vacant and abandoned lots and other uninhabited areas were at higher risk for DENV infections since these properties supported early-stage *A. aegypti* development within containers of still water (Barrera et al., 2021). In Colombia, locations closer to, and with a greater utilization of, public transit recorded higher DENV case counts (Shragai et al., 2022). Living near junk yards, tire repair shops, and deposits of recyclable materials was also closely associated with increased DENV incidence in Brazil (Johansen et al., 2018). Living near a health unit in the community was associated with higher DENV infection risk since these increased opportunities for case detection (Kikuti et al., 2015; Kalbus et al., 2021). Other sociocultural community patterns shared between members of the community are also crucial social factors. For instance, Stewart Ibarra et al. (2013) discovered that households that shared their property with other households were at greater risk of DENV infection since sharing a common space, such as a patio, may affect community members’ water storage practices, thereby creating prolific vector breeding sites.

#### Household-Level Factors

Structural deficiencies of houses were associated with DENV transmission since they may provide pathways for mosquito access into the household environment (Lippi et al., 2018; Campos et al., 2021). For instance, those who lived in *ranchos* (i.e., shacks, informal housing) in Venezuela were nearly seven times more likely to have had a previous DENV infection than those living in houses with better conditions (Velasco-Salas et al., 2014). Similar findings were documented from Brazilian *favelas* (Flauzino et al., 2009). Maccormack-Gelles et al. (2018) reported that higher average annual household income was strongly associated with reduced DENV incidence in Fortaleza, Brazil. Spatial modeling studies on DENV incidence corroborated these findings (Teixeira & Cruz, 2011; Kikuti et al., 2015). Other important household infrastructural determinants of DENV infection included interruptions to household piped water supply since inadequate water supply promotes water-storing behaviors and practices (Quintero et al., 2009). Using air conditioners to cool the home was negatively associated with *A. aegypti* abundance in Ecuador since it reduced the need for opening windows and doors in the house (Lippi et al., 2021). Households with toilets without direct discharge were associated with higher DENV risk and increased the number of potential breeding sites for vectors (Martínez-Vega et al., 2015).

Household compositional characteristics related to dengue outcomes included household population density (i.e., number of household members), where households with more residents increased the probability of infectious bites and DENV exposure and risk (Vincenti-Gonzalez et al., 2017). Living in households with fewer rooms, and households with children under the age of 5, were also associated with an increased risk of DENV infection and seropositivity (Martínez-Vega et al., 2015). House ownership, as opposed to non-ownership, was associated with decreased DENV incidence since residents of not-owned households may have had a weaker sense of belonging to the house, which could represent a diminished cleanliness of the house and its surroundings (Johansen et al., 2018). Both recent and past DENV infections were positively associated with the number of years lived in the same residence (Velasco-Salas et al., 2014). Lack of access to electricity and Internet within households was also associated with poorer DENV-related outcomes since access to Internet and basic electric utilities was linked with access to sources of knowledge, better living conditions, and overall positive preventive behaviors (Vásquez-Trujillo et al., 2021).

#### Individual-Level Factors

Several studies found no association between sex and gender and DENV outcomes, and findings generally reported that both sexes were equally affected (Carabalí et al., 2017; Chiaravalloti-Neto et al., 2019). However, in Amazonia, rates of DENV infections were higher among men than women (da Silva-Nunes et al., 2008), whereas in Peru, higher rates of infection were found among women compared to men (Charette et al., 2020). The type of occupation and individual’s employment status was a significant predictor of recent DENV infection in Venezuela, where people who spent more time within homes, such as domestic workers and housewives, were at a higher risk of contracting DENV (Velasco-Salas et al., 2014; Vincenti-Gonzalez et al., 2017). Lower levels of educational attainment and literacy were associated with DENV infection in Brazil (da Conceição Araújo et al., 2020) and Ecuador (Lippi et al., 2018). Ferreira et al. (2021) found that individuals with lower levels of educational attainment were among those with lower levels of knowledge about DENV. Misconceptions about DENV still exist in LAC (Stewart Ibarra et al., 2014). Knowledge, attitudes, and practices (KAP) studies showed that adequate knowledge about DENV transmission methods, and the appropriate attitudes and use of preventative measures, have shown a protective effect against DENV infection (Benítez-Díaz et al., 2020). However, some studies have implied that DENV-related KAP does not necessarily translate into human behavioral changes that consolidate recommended prevention practices (Shuaib et al., 2010). Additionally, low individual-level socioeconomic status (SES) was associated with higher rates of DENV infection (Braga et al., 2010; Farinelli et al. 2018). Bavia and colleagues (2020) found a statistically significant negative relationship between mean income, which is a strong indicator of SES and DENV incidence in southern Brazil.

## Discussion

### Overview of the Current Evidence

Antecedent efforts, largely based on research from the Special Programme for Research and Training for Tropical Diseases (TDR) at the World Health Organization (WHO), funded by the Ecosystems and Human Health (Ecohealth) Programme at Canada’s International Development Research Centre (IDRC), identified useful steps for investigating the ecological, biological, and social determinants of dengue and other vector-borne infectious diseases, and inspired the present work. We reviewed the literature to compile the evidence for the distinct ecological, biological, and social determinants of DENV vector dynamics, transmission, and epidemiology in LAC. Key findings from this review provided constructive evidence for recognizing the contributing factors of dengue emergence and re-emergence within LAC over the last 50 years.

The role of environmental and ecological determinants such as meteorological, climatological, and seasonal factors in influencing patterns of dengue transmission was emphasized in the literature. Particularly, there was a strong and significant relationship between indices of temperature and precipitation and the occurrence of dengue epidemics. Measures of relative humidity revealed similar effects as temperature on DENV occurrence since at higher temperatures; the air may contain more water vapor than the same volume of air at lower temperatures (Carneiro et al., 2017). For this reason, higher humidity levels at ideal temperature parameters can determine DENV transmission potential by regulating the location and magnitude of DENV risk (Campbell et al., 2015). The relationship between these factors and dengue outcomes is mediated by conductive changes to the life cycle of the DENV vector (e.g., breeding site availability; Kraemer et al., 2015).

The effect of artificial environmental factors that mediate inner-city temperatures, such as the UHI effect and pollution, on dengue transmission dynamics was addressed briefly in the literature. Urban areas are complex mosaics of heterogeneous environmental conditions that provide an epidemiological landscape for the DENV transmission chain (Pickett et al., 2011). More research in this field is needed to elucidate the physiographic (i.e., architectural) and territorial characteristics of the artificial environment that moderate dengue occurrence. Studies on global scale environmental factors such as climate change and the ENSO phenomenon and its impact on dengue occurrence at regional and local scales were limited. Against a backdrop of a warming globe, climatological factors (i.e., seasonal weather patterns at long-term time scales) have become unpredictable, and dramatic alterations to seasonal macroclimatic weather oscillations have been evidenced in LAC as a result (e.g., increased warm temperatures, rise in maximum sea levels, increased rainfall, etc.; IPCC, 2014). These factors may be associated with increasing the ecological suitability and plasticity of DENV vectors and the expansion of DENV risk in novel geographic settings (Naish et al., 2014).

We identified several biological determinants of dengue outcomes in the literature, including the impact of genetic variabilities on vectorial capacity and competence. Laboratory-based studies demonstrated the potential for *A. aegypti* populations from novel regions to transmit dengue (Lourenço de Oliveira et al., 2013). Vector competition and serotype dynamics were found to play a significant role in the dengue system, with implications for disease transmission patterns. Pathogenic indicators of the virus constituted biological determinants, and phylogeographic analyses demonstrated that the transmission of DENV serotypes vary at geographic and temporal scales. Other genetic studies demonstrated that the dengue viruses are competing and evolving, often through the deleterious genetic mutations of the virus and the progressive elimination of a particular lineage through genetic negative selection (i.e., purifying selection). This selection may depend on specific genotype-by-genotype (G × G) interactions, where vectorial capacity can determine serotype dominance and infection outcomes (Lambrechts et al., 2009). Importantly, serotype competition, and the resultant dominance of individual serotypes among human populations, may be influenced by the immunological profiles due to cross-reactive humoral and cellular immune responses between serotypes (Reiner Jr. et al., 2014).

We summarized the influence of social determinants on dengue transmission at global, community, household, and individual levels. Globalization facilitates DENV disease emergence and re-emergence in the Americas. International movement and migration of humans and vectors were identified as key drivers of dengue transmission in the region. Community and household-level factors were the most cited determinants of dengue outcomes in this review, including the location of the community relative to shared public spaces and transportation lines, and the structural conditions, infrastructure, and sanitation of households. Household compositional characteristics effectively determined DENV exposure and risk of household residents, underscoring the importance of integrated approaches to vector control and community engagement. Individual-level factors such as occupation, education, and knowledge about dengue were found to shape vulnerability to infection and exposure to DENV. The relationship between socioeconomic and cultural background and dengue infection, disaggregated by gender, should be assessed in future studies for the development of targeted interventions tailored to specific population groups. Importantly, poverty is a determinant of determinants, where unfavorable socioeconomic conditions are associated with lower levels of income, educational attainment and literacy, unemployment, and inadequate access to health services, among other barriers to health, and may mediate the relationship between SES and dengue epidemiology in LAC.

## Limitations

One limitation of this review is the reliance on previously published research and the availability of these studies using the search strategy. Second, while several systematic methods and tools were used to conduct this review, this work is presented as a review and not a systematic review. Third, the heterogeneity of study methodologies and designs may have limited the comparability of findings across studies and LAC sub-regions. Fourth, this review emphasized the role of *A. aegypti* in shaping dengue outcomes and understated the role of the *A. albopictus* vector. We focused on the urban cycle of DENV transmission where the *A. aegypti* mosquito is more prevalent in the study region.

## Conclusions

To the best of our knowledge, no study has compiled the existing evidence for the ecological, biological, and social determinants of dengue epidemiology in LAC. In this review, we underscored the multifaceted nature of dengue transmission dynamics in LAC. This scoping review serves as a starting point for future research to equip policymakers and public health practitioners toward developing effective strategies to mitigate the impact of dengue and protect vulnerable populations in the region. Prospective research should include qualitative research that investigates the differential experiences of communities and individuals and explores the latent, personalized social and cultural factors involved in DENV transmission. Future works may also endeavor to investigate the dynamic relationships between all the climatological, meteorological, and geographical; pathological and entomological; and socioeconomic, demographic, and cultural factors that regulate DENV vector dynamics and DENV transmission and epidemiological outcomes in LAC.

## Supplementary Information

Below is the link to the electronic supplementary material.

Supplementary file1 (DOCX 30 KB)
